# Synthesis, Characterization, and Toxicity Assessment of Zinc Oxide-Doped Manganese Oxide Nanoparticles in a Macrophage Model

**DOI:** 10.3390/ph17020168

**Published:** 2024-01-29

**Authors:** Nasser B. Alsaleh, Anas M. Aljarbou, Mohamed E. Assal, Mohammed A. Assiri, Mohammed M. Almutairi, Homood M. As Sobeai, Ali A. Alshamrani, Sultan Almudimeegh, Mohammad R. Hatshan, Syed F. Adil

**Affiliations:** 1 Department of Pharmacology and Toxicology, College of Pharmacy, King Saud University, P.O. Box 2457, Riyadh 11451, Saudi Arabia; 442106265@student.ksu.edu.sa (A.M.A.); moassiri@ksu.edu.sa (M.A.A.); malmotyre@ksu.edu.sa (M.M.A.); hassobeai@ksu.edu.sa (H.M.A.S.); aaalshamrani@ksu.edu.sa (A.A.A.); salmudimeegh@ksu.edu.sa (S.A.); 2 Department of Chemistry, College of Science, King Saud University, P.O. Box 2455, Riyadh 11451, Saudi Arabia; masl@ksu.edu.sa (M.E.A.); mhatshan@ksu.edu.sa (M.R.H.)

**Keywords:** nanomaterials, doping, toxicity, immune cell

## Abstract

The doping of engineered nanomaterials (ENMs) is a key tool for manipulating the properties of ENMs (e.g., electromagnetic, optical, etc.) for different therapeutic applications. However, adverse health outcomes and the cellular biointeraction of doped ENMs, compared to undoped counterparts, are not fully understood. Previously, we have shown that doping manganese oxide nanoparticles with ZnO (ZnO-MnO_2_ NPs) improved their catalytic properties. In this study, we assessed the toxicity of ZnO-MnO_2_ NPs in Raw 264.7 cells. NPs were prepared via an eco-friendly, co-precipitation method and characterized by several techniques, including transmission and scanning electron microscopy, X-ray diffraction, and Fourier transform infrared. The physicochemical properties of ZnO-MnO_2_ NPs, including size, morphology, and crystalline structure, were almost identical to MnO_2_ NPs. However, ZnO-MnO_2_ NPs showed slightly larger particle aggregates and negative charge in cell culture media. Exposure to ZnO-MnO_2_ NPs resulted in lower toxicity based on the cell viability and functional assay (phagocytosis) data. Exposure to both NPs resulted in the activation of the cell inflammatory response and the generation of reactive oxygen species (ROS). Despite this, exposure to ZnO-MnO_2_ NPs was associated with a lower toxicity profile, and it resulted in a higher ROS burst and the activation of the cell antioxidant system, hence indicating that MnO_2_ NP-induced toxicity is potentially mediated via other ROS-independent pathways. Furthermore, the cellular internalization of ZnO-MnO_2_ NPs was lower compared to MnO_2_ NPs, and this could explain the lower extent of toxicity of ZnO-MnO_2_ NPs and suggests Zn-driven ROS generation. Together, the findings of this report suggest that ZnO (1%) doping impacts cellular biointeraction and the consequent toxicological outcomes of MnO_2_ NPs in Raw 264.7 cells.

## 1. Introduction

Engineered nanomaterials (ENMs), precisely manmade materials within the nanoscale (1–100 nm), are being developed for various applications across different industrial sectors [1]. Given the vast potential for the tunability of inorganic ENM physicochemical properties (e.g., optical, electromagnetic, etc.), there has been an increase in the development of inorganic ENMs over the past years, including biomedical applications, such as nanomedicine and tissue engineering [2,3]. As a result, human exposure to ENMs, including intentional exposure such as in the case of nanomedicine, will be unavoidable. However, to date, there remains uncertainty regarding the safety profile and potential toxicological manifestations associated with exposure to ENMs [4,5].

The area of material science with a focus on synthesizing nanocomposites of different inorganic materials or metal-organic frameworks has gained attention over the past few years [6,7]. The doping of inorganic ENMs, which is the intentional introduction of a trace of inorganic material into the crystal lattice of inorganic ENMs, is a key tool for manipulating ENM physicochemical properties (e.g., electromagnetic, photocatalytic, optical, etc.) towards desired applications [8]. Indeed, previous efforts have demonstrated that the doping of inorganic ENMs may increase cytotoxicity against cancer cells [9], improve antimicrobial properties [10], enhance magnetic properties [11], and increase the capacity for drug loading and delivery [12]. Despite doping’s potential in improving the biomedical applications of ENMs, there remains uncertainty and inconsistency in the literature with regard to the impact of doping on ENM toxicological outcomes. Currently, there is inconsistency with regard to the toxicological outcomes of doped materials as doping has been shown to influence ENM toxicity both positively and negatively [13,14]. In addition, the therapeutic applications of ENM doping are often pursued without enough assessment of the potential toxicity [14]. Therefore, there is a need for more research to improve our understanding of the nature of cellular biointeractions and the toxicity of doped ENMs in comparison with their undoped counterparts [15].

Manganese is an essential micronutrient for basic cell biochemical reactions involved in growth, differentiation, and homeostasis [16]. In the past few years, manganese oxide nanoparticles (MnO_x_ NPs) have attracted attention for applications across multiple disciplines including catalysis, solar cells, and water treatment, as well as in biomedical applications including bioimaging and biosensors, drug delivery, and cancer theranostics [17,18,19,20]. Due to the high tunability of their physicochemical properties (e.g., size, shape, surface functionalization, etc.), low cost and high abundancy, the sustainability of synthesis via green methods, biocompatibility, and low toxicity profile, MnO_x_ NPs represent an excellent material for a wide range of biomedical applications [17]. It is also recognized that a key attractive property of using MnO_x_ NPs in cancer therapy stems from acting as a transition metal and hence participating in Fenton-like reactions to generate free radicals [21,22,23]. Previous reports demonstrated in vivo toxicity following exposure to MnO_x_ NPs with increased toxicity outcomes in comparison to bulk materials [24,25,26]. An important toxicological aspect to consider is the biodegradability of ENMs [27]. Indeed, a major drawback of numerous inorganic ENMs with key biomedical applications (e.g., gold, silver, silica, etc.) is their biopersistence [28]. More studies are now focused on synthesizing biodegradable materials, as well as understanding the underlying mechanisms of biodegradability, including Mn-based ENMs [28,29,30,31].

Currently, there is a growing interest in exploiting and fine-tuning the redox properties of MnO_x_ NPs via doping with other metals [32,33,34]. Nevertheless, the safety profile and toxicological outcomes of such platforms are yet to be studied. We have previously shown that doping MnO_x_ NPs with ZnO improved their catalytic properties, where doping with ZnO (1%) resulted in the highest catalysis [35]. Here, we investigated whether doping with ZnO (1%) could affect the toxicity outcomes of MnO_2_ NPs in Raw 264.7 cells, an established macrophage-like model. To our knowledge, this is the first report assessing the toxicity of ZnO-doped MnO_2_ NPs and their toxicological consequence.

## 2. Results and Discussion

### 2.1. Nanoparticle Characterization

**Figure 1:**
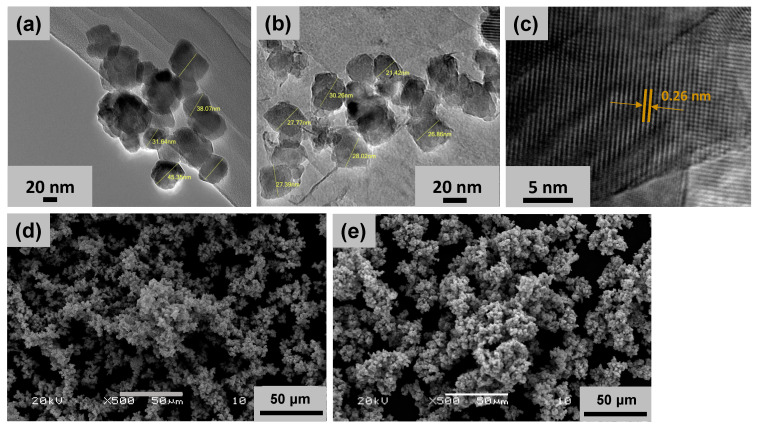
Representative transmission and scanning electron microscopy (SEM and TEM) images of the nanoparticles. Representative TEM images of (**a**) MnO_2_ NPs and (**b**) ZnO-MnO_2_ NPs were obtained after dispersion of the NPs via ultrasonication before microscopy analysis. (**c**) TEM image of ZnO-MnO_2_ NPs at higher magnification showing interplanar lattice fringes of 0.26 nm (arrows). Representative SEM images of (**d**) MnO_2_ NPs and (**e**) ZnO-MnO_2_ NPs.

The synthesis of MnO_2_ NPs and ZnO-MnO_2_ NPs was carried out through an eco-friendly, facile and straightforward co-precipitation procedure, with calcination at 400 °C in a muffle furnace to obtain the desired NPs. The crystallinity, composition, and morphology of fabricated NPs were assessed using XRD, TGA, FT-IR, EDX, SEM, and HR-TEM. The surface morphology of the fabricated NPs was studied using TEM and SEM. The representative images showed that both NPs have similar particle shapes (Figure 1). The HR-TEM images of ZnO-MnO_2_ NPs displayed well-defined nano-sized polycrystalline particles with evident interplanar lattice fringes of 0.26 nm that are related to the plane (2 0 1) of MnO_2_ NPs, whereas the morphology of the MnO_2_ NPs was not well defined according to the TEM images (Figure 1).

**Figure 2:**
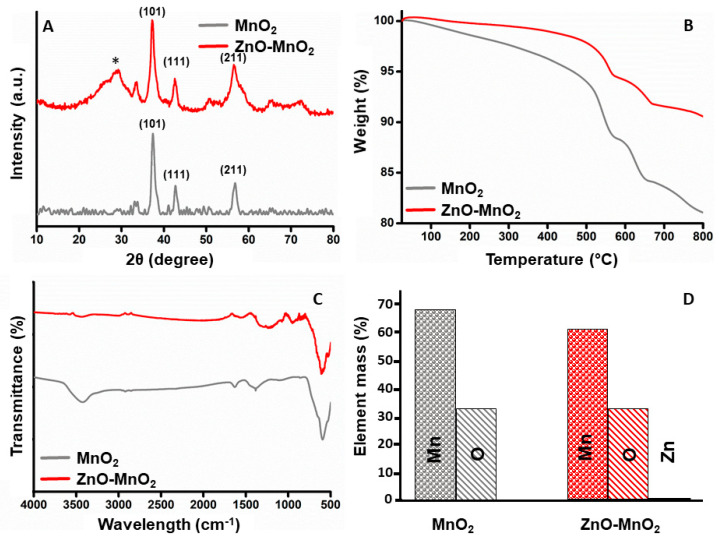
Characterization of nanoparticles. (**A**) Comparative X-ray diffractogram for the MnO_2_ NPs (grey line) or ZnO-MnO_2_ NPs (red line) scanned between 2θ 10 and 80 (* indicates diffraction signal); (**B**) results from the thermogravimetric analysis of MnO_2_ NPs (grey line) or ZnO-MnO_2_ NPs (red line) in the range from 25 °C to 800 °C; (**C**) Fourier transform infrared spectrum of MnO_2_ NPs (grey line) or ZnO-MnO_2_ NPs (red line) in the range from 400 cm^−1^ to 4000 cm^−1^; (**D**) graphical illustration of elemental analysis of the MnO_2_ NPs (grey) or ZnO-MnO_2_ NPs (red).

The crystal structure of the NPs was assessed using XRD analysis. The XRD patterns for the synthesized MnO_2_ NPs and ZnO-MnO_2_ NPs are presented in Figure 2A. The obtained diffraction peaks distinctly show that the fabricated materials are crystalline in nature. The MnO_2_ NPs annealed at 400 °C exhibited a pattern that is highly in accordance with the standard XRD results of pyrolusite manganese dioxide (JCPDS-No. 24-0735). As anticipated, ZnO-MnO_2_ NPs showed a similar XRD pattern to that of MnO_2_ NPs, in addition to the diffraction signal labelled with star (*), which is possibly ascribed to the existence of ZnO NPs. The thermal properties of the synthesized NPs were examined using thermal gravimetric analysis (TGA) (Figure 2B). The TGA graph of MnO_2_ NPs demonstrated thermal stability with overall weight loss around 18% and up to 800 °C, while for the ZnO-MnO_2_ NPs, it was observed that total weight loss was only about 8% at 800 °C, indicating that ZnO-MnO_2_ NPs is more thermally stable than pure MnO_2_ NPs. The FT-IR analysis was employed to identify the surface functionalities of the as-obtained materials, and the results of the synthesized MnO_2_ NPs and ZnO-MnO_2_ NPs were demonstrated in Figure 2C. The FT-IR peak located at nearly 3425 cm^−1^ and 1640 cm^−1^ is associated with the characteristic vibrations of hydroxyl groups [36]. In addition, the intense peaks at 602 cm^−1^ are associated with the characteristic of Mn–O vibrations [37,38]. The EDX plot of the synthesized ZnO-MnO_2_ NPs and MnO_2_ NPs is shown in Figure 2D. The left-side EDX figure demonstrates the mass percentage composition of Mn and O elements in MnO_2_ NPs. The right side illustrates the EDX plot of the synthesized ZnO-MnO_2_ NPs, which indicated that the values obtained for the Mn and O composition elements are similar to the values obtained for MnO_2_ NPs, along with the appearance of a small peak representing the amount of Zn, hence reflecting the successful inclusion of ZnO NPs in the system [35]. The values in tabular format are shown in Table S1 in the Supplementary Materials.

### 2.2. Nanoparticle Preparation for In Vitro Studies and Characterization in Vehicle and Cell Culture Media (DMEM)

For in vitro experiments, NP stocks were prepared in ultrapure water (UPW; vehicle) and then diluted in serum-free cell culture media. Both NPs demonstrated larger hydrodynamic size (d_h_), an indication of particle aggregation and/or agglomeration (Table 1). This is typically observed in nanotoxicological studies, particularly for uncoated inorganic NPs. Nevertheless, the polydispersity indices (PDIs) of both NP types in both UPW and DMEM were equal to or less than 0.3 (Table 1). The suspended MnO_2_ NPs had a hydrodynamic size of 197 ± 35 (d_h-UPW_) and 483 ± 15 in cell culture media (d_h-DMEM_) while ZnO-MnO_2_ NPs had a hydrodynamic size of 245 ± 7 in UPW (d_h-UPW_) and 546 ± 30 in cell culture media (d_h-DMEM_) (Table 1). The zeta potential (ζ) of the MnO_2_ NPs was −16.2 ± 3.7 in UPW (ζ_UPW_) and −4.8 ± 1 in cell culture media (ζ_DMEM_), while for the ZnO-MnO_2_ NPs, it was −20 ± 5.2 in UPW (ζ_UPW_) and −5.5 ± 1.8 in cell culture media (ζ_DMEM_) (Table 1).

### 2.3. Cell Viability following Nanomaterial Treatment

The toxicity of MnO_x_ NPs has been previously reported by several studies [24,25,26]. For instance, an early study showed that 28-day oral repeated exposure to MnO_2_ NPs in Wistar rats was associated with larger tissue distribution and increased toxicity (e.g., DNA damage, biochemical and histopathological alterations, etc.) in comparison with bulk MnO_2_ [24]. Another study has shown that exposure of Wistar rats to MnO_2_ NPs via intratracheal instillation for 6 weeks resulted in size-dependent toxicity, with the smaller NPs resulting in more pronounced toxicity [26]. Together, these reports suggest that exposure to MnO_x_ NPs may induce toxicity, particularly following chronic exposure [24,25,26]. However, the toxicity and biointeraction of MnO_x_ NPs at cellular and molecular levels remain largely unknown. It is worth mentioning that previous works have exploited the property of MnO_x_ NPs to induce Fenton-like reactions and generate ROS (e.g., platforms used in photodynamic therapy) [21,22,23]. Such studies suggest that the toxicity of MnO_x_ NPs is largely driven via ROS generation, leading to oxidative stress and cellular damage. However, such a hypothesis is yet to be validated, and the safety profile for such platforms on normal cells remains to be studied.

**Figure 3:**
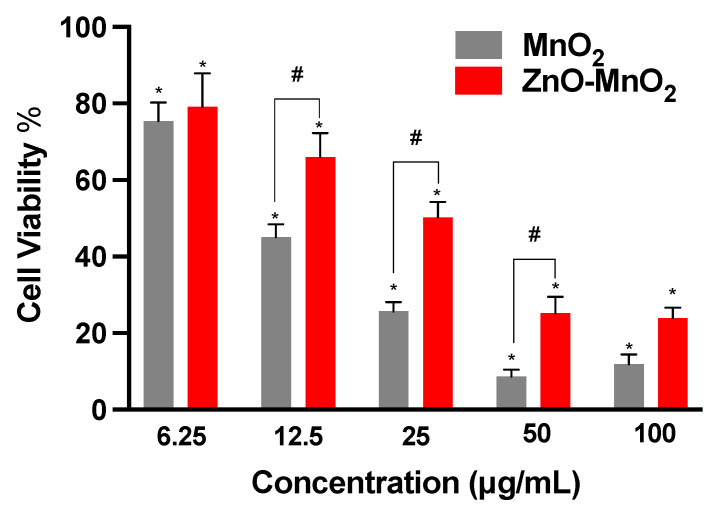
Viability of Raw 264.7 cells following exposure to nanoparticles. Cellular viability was estimated based on the formation of formazan salt after 24 h exposure to MnO_2_ NPs (grey bars) or ZnO-MnO_2_ NPs (red bars) within a range of 6.25–100 μg/mL in serum-free media. Independent experiments were carried out at least 3 times (*n* ≥ 3). A *p* value of less than 0.05 (*p* < 0.05) indicates statistical significance between control and treatment groups (*) or between treatment groups (#).

Here, the data indicated concentration-dependent toxicity after treatment with both MnO_2_ NPs and ZnO-MnO_2_ NPs (Figure 3). However, the reduction in cell viability was significantly lower following exposure to ZnO-MnO_2_ NPs in comparison with undoped MnO_2_ NPs at several concentration points (12.5, 25, and 50 μg/mL). It is worth noting that ZnO-MnO_2_ NPs appeared to have a tendency to form larger agglomerates and/or aggregates and have larger negative charge upon suspension in cell culture media (DMEM). Such formation of NP agglomerates and/or aggregates typically occurs with metal and metal oxide ENMs, particularly those with no or minimal surface coating [39,40,41]. Despite the fact that changes in hydrodynamic size and surface charge after doping were within a relatively close range, the impact on toxicity cannot be ruled out. However, other factors (e.g., Mn ion release) might have been influenced by doping and contributed to the observed difference in toxicity. Indeed, a previous report has demonstrated that ZnO-doped TiO_2_ NPs (1, 5, and 10%) did not influence the hydrodynamic size or surface charge, yet they had a significant impact on toxicity. However, the toxicity was potentially mediated via the release of dopant ions [9]. Based on viability data, a dose of 12.5 μg/mL was used throughout subsequent experiments.

### 2.4. Cell Functional Competence following Nanomaterial Treatment

**Figure 4:**
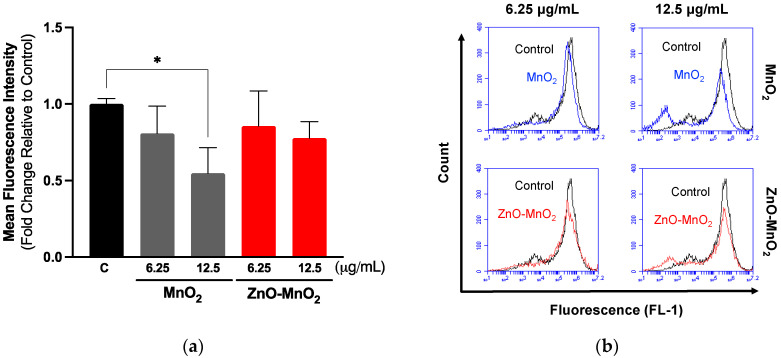
Functional capacity of Raw 264.7 cells following exposure to nanoparticles. (**a**) Cell phagocytosis capacity was estimated based on the internalization of fluorescently labeled latex beads (4 h) following exposure to 6.25 or 12.5 μg/mL of MnO_2_ NPs (grey bars) or ZnO-MnO_2_ NPs (red bars) for 2 h. (**b**) Representative histograms of the data from the flow cytometer. Independent experiments were carried out at least 3 times (*n* ≥ 3). A *p* value of less than 0.05 (*p* < 0.05) indicates statistical significance between control and treatment groups (*).

Measuring cell functional competence following exposure to ENMs is an important parameter in the safety assessment of ENMs [42,43]. Macrophage is a key cell type for the elimination of invading pathogens and foreign bodies, including ENMs [44]. They are also a key component in engaging adaptive immunity [44]. Ample evidence has demonstrated the detrimental consequences of ENMs on macrophage health and function [45,46]. Earlier studies have demonstrated that treatment with even subtoxic levels of ENMs could result in adverse consequences with respect to macrophage function [43,46,47,48]. As part of assessing NP safety in Raw 264.7 cells, we measured cell phagocytic capacities based on the uptake of fluorescently labeled beads following exposure to the NPs for 2 h, followed by the washing of NPs and incubation with fluorescently labeled beads for 4 h. It is worth noting that the exposure time was shortened to prevent overwhelming the cell’s functional capacity while the dose was kept at the same concentration points as in the viability experiments. The data demonstrated that exposure to both types of NPs was associated with a dose-dependent trend of toxicity relative to the cell capacity for phagocytosis (Figure 4). However, exposure to MnO_2_ NPs resulted in a larger suppression of the cell’s phagocytic capacity compared to ZnO-MnO_2_ NPs. These results are in line with the viability data indicating that ZnO doping ameliorated the toxicity of MnO_2_ NPs in Raw 264.7 cells.

### 2.5. Inflammatory Response and Reactive Oxygen Species Generation following Nanomaterial Treatment

**Figure 5:**
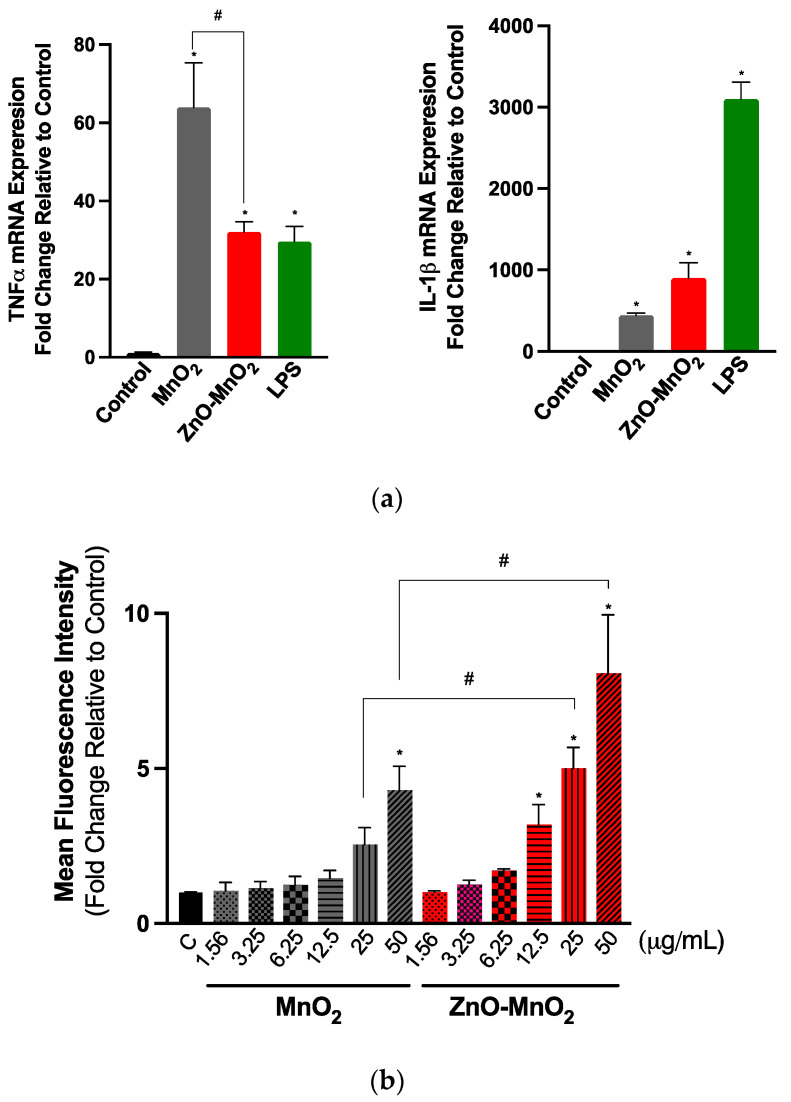
Inflammatory response and reactive oxygen generation following exposure to the nanoparticles. (**a**) *TNF-α* (left panel) and *IL-1β* (right panel) gene expression following exposure to NPs for 6 h. (**b**) The generation of intercellular ROS was assessed based on the fluorescence signal of dichlorofluorescin diacetate (H2DCFDA) following exposure to a range of concentrations (1.56–50 μg/mL) of MnO_2_ NPs (grey bars) or ZnO-MnO_2_ NPs (red bars) for 60 min. Treatment with lipopolysaccharide (LPS) (1 µg/mL) was included for comparison purposes. Independent experiments were carried out at least 3 times (*n* ≥ 3). A *p* value of less than 0.05 (*p* < 0.05) indicates statistical significance between control and treatment groups (*) or between treatment groups (#).

The activation of cell inflammatory pathways is a critical cellular response to toxic insults [49]. It is important for overcoming stress, and it is part of the repair mechanism following exposure to environmental insults, including ENMs [49]. It also represents *Tier-2* of the previously proposed “hierarchical oxidative stress” model following exposure to ENMs [50]. The data of this study suggest that exposure to both NPs activated the inflammatory response, including the expression of *TNFα* and *IL-1β* genes (Figure 5a). Exposure to ZnO-MnO_2_ NPs had a lower activation of *TNFα* compared to MnO_2_ NPs. However, ZnO-MnO_2_ NPs appeared to have a stronger activation of *IL-1β*. These results may suggest that doping with ZnO might have altered MnO_2_ NP-mediated cellular interaction and biological outcomes. However, more work is needed to confirm such a hypothesis. For instance, the screening of a panel of inflammatory markers (e.g., pro- vs. anti-inflammatory) based on cytokine release will help delineate the impact of NPs on inflammatory pathways (i.e., doped vs. undoped).

A major paradigm of ENM toxicity is driven via the ENM-induced generation of reactive oxygen species (ROS), eventually leading to oxidative damage [50]. MnO_x_ NPs have been shown to generate ROS by acting as a transition metal, resulting in Fenton-like reactions [21,22,23]. The doping and intentional introduction of chemical defects into metal oxide ENMs have been successfully utilized to manipulate their properties towards a wide range of biomedical applications [8,15]. The unintentional formation of chemical defects could also result during the large-scale production of ENMs [13]. From a toxicological perspective, earlier research has established that the doping of metal oxide ENMs can lead to changes in (i.e., narrowing or expansion) their band gap and associated electron transfer to cellular redox species (i.e., the overlap between intracellular redox potential and ENM energy bands) and, hence, the generation of ROS and subsequent oxidative stress-mediated toxicity [51,52]. Indeed, a previous report showed that chemical defects present in ZnO NPs contributed to their cellular toxicity via NP–protein charge interaction or NP-induced ROS generation [53]. Nevertheless, doping could also ameliorate the toxicity of NPs via other mechanisms, such as increasing the material stability and reducing dissolution and the release of ions, which have been shown to contribute largely to the toxicity of some NPs, such as ZnO and CuO [54,55]. Our results indicated that exposure to NPs for 60 min led to ROS burst (Figure 5b). However, ROS levels were significantly higher in cells treated with ZnO-MnO_2_ NPs. This was to our surprise since MnO_2_ NPs resulted in increased toxicity; hence, we anticipated that it would induce higher levels of ROS compared to doped NPs. Although we did not measure the NP band gap in this study, the possibility of a change in the band gap upon doping cannot be ruled out. However, even in the case of a change in the NP band gap and associated ROS generation, the toxicity of undoped MnO_2_ NPs was significantly higher than ZnO-doped MnO_2_ NPs. This suggests that exposure to undoped MnO_2_ NPs induced toxicity through other ROS-independent pathways. Indeed, the previous literature has shown that ENMs could induce toxicity relative to mammalian and eukaryotic cells via mechanisms that are independent of ROS generation. These mechanisms include the ROS-independent induction of cell cycle arrest, apoptosis, mitochondrial damage (respiratory chain complex IV), autophagy, and inflammatory cytokines [56,57,58,59]. Studies have also demonstrated a role for ENM concentration in mediating ROS-dependent versus -independent toxicity [58,59].

### 2.6. Antioxidant Response following Nanomaterial Treatment

**Figure 6:**
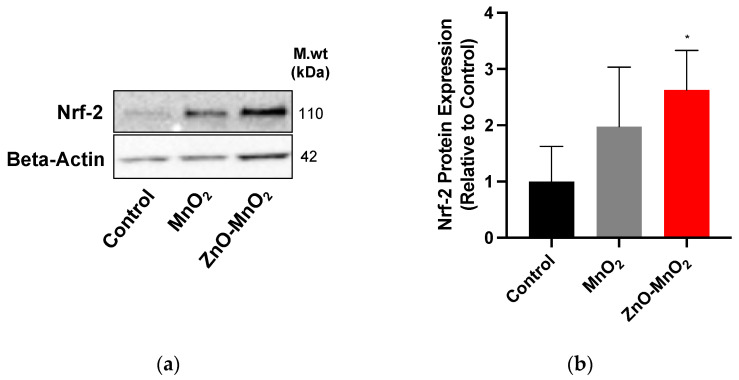
Antioxidant response following exposure to nanoparticles. (**a**) Nrf-2 protein expression representative blot and (**b**) the quantification of Western blots after treatment with MnO_2_ NPs (grey bars) or ZnO-MnO_2_ NPs (red bars) (12.5 µg/mL) for 24 h. Independent experiments were carried out at least 3 times (*n* ≥ 3). A *p* value of less than 0.05 (*p* < 0.05) indicates statistical significance between control and treatment groups (*).

We measured the protein expression of nuclear factor-erythroid 2-related factor 2 (Nrf-2), a central regulator of the cell antioxidant system, following exposure to NPs to assess the activation of the cell antioxidant system. Consistent with ROS data, exposure to ZnO-MnO_2_ NPs appeared to induce the higher expression of Nrf-2 proteins compared to MnO_2_ NPs (Figure 6). Together, the data indicate that doping with ZnO led to the generation of more ROS and the consequent activation of the cell antioxidant system, as evidenced by the higher expression of Nrf-2.

### 2.7. Cellular Internalization following Nanomaterial Treatment

**Figure 7:**
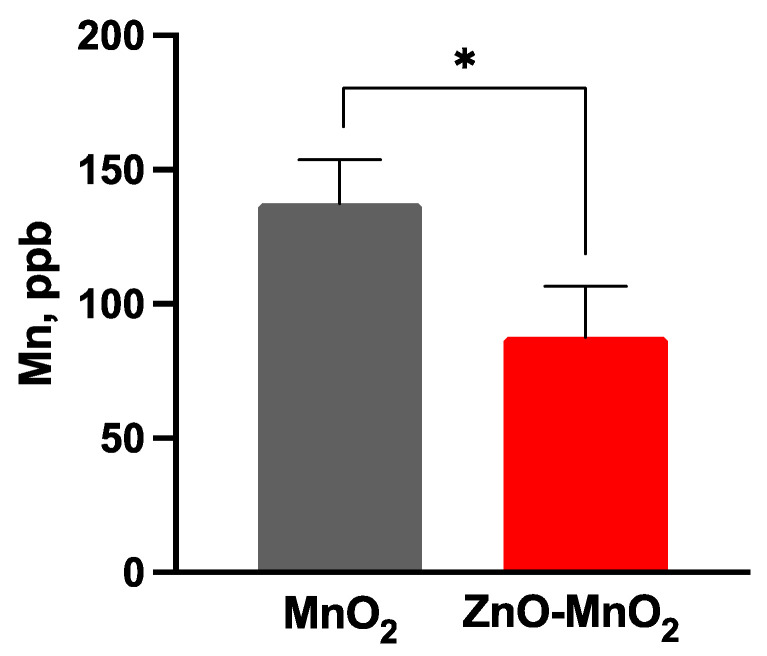
Nanoparticle internalization following exposure to nanoparticles. The cell internalization of the nanoparticle was measured based on the Mn content following treatment with MnO_2_ NPs (grey bars) or ZnO-MnO_2_ NPs (red bars) (12.5 μg/mL) for 24 h. Independent experiments were carried out at least 3 times (*n* ≥ 3). A *p* value of less than 0.05 (*p* < 0.05) indicates statistical significance between control and treatment groups (*).

The cellular internalization of ENMs is one of the key parameters in the assessment of ENM safety. We measured NP internalization via ICP-MS, a gold standard technique for measuring metal content. The data indicated that treatment with ZnO-MnO_2_ NPs resulted in a significant reduction in NP internalization compared to MnO_2_ NPs based on the Mn content (i.e., a difference of approximately 50% ppb between the doped and undoped NPs at 12.5 µg/mL) (Figure 7). Importantly, our data also showed increased cellular Zn content following exposure to the doped NPs compared to their undoped control (Figure S1). This could suggest the release of Zn ions that had subsequently generated ROS and influenced the cell molecular response (e.g., ROS generation and inflammatory pathways) as previously reported [9]. However, unlike the reported data where Zn doping increased toxicity, our findings showed less toxicity following exposure to ZnO-doped MnO_2_ NPs. A possible explanation for the observed data is that doping in our case might have influenced the cellular internalization of NPs, interaction with and damage of intracellular organelles, and/or release of Mn ions. The cellular internalization of ENMs is a key parameter for understanding their biointeraction. ENM toxicity is often driven via cellular internalization, regardless of whether it is a result of active or passive cellular uptake (e.g., cell membrane damage) [60,61,62]. Subsequently, toxicity could then arise from lysosomal-mediated ion release, interactions with cellular organelles and the generation of ROS, and consequent macromolecular damage and disruption of organelle function [60,61]. Currently, it is hard to explain such differences in cellular internalization since the hydrodynamic size and surface charge of both NPs were within a close range; however, the impact of the relatively larger hydrodynamic size and smaller negative charge of the doped NPs on their cellular internalization cannot be ruled out. Further studies, such as studying multiple doping contents (e.g., 1% vs. 5% vs. 7%, etc.) and their impact on hydrodynamic size and surface charge, are needed to understand the impact of doping on NP physicochemical properties, cellular internalization, and toxicity.

## 3. Materials and Methods

### 3.1. Nanoparticle Synthesis and Characterization

ZnO-MnO_2_ NPs were synthesized using an eco-friendly and facile co-precipitation procedure. Stoichiometric amounts of manganese(II)nitrate-tetrahydrate (Mn(NO_3_)_2_·4H_2_O) and zinc nitrate hexahydrate (Zn(NO_3_)_2_·6H_2_O) were dissolved in distilled water. The mixture of solutions of Mn(NO_3_)_2_·4H_2_O and Zn(NO_3_)_2_·6H_2_O (100 mL) was stirred and transferred to a three-neck round-bottomed flask. The resulting solution was heated to 90 °C and vigorously stirred using a magnetic bar, and a solution of NaHCO_3_ with a concentration of 0.50 M was added dropwise until the pH value of the solution reached around 9. The solution was continuously stirred at the same temperature of 90 °C for 3 h, and stirring occurred overnight at room temperature. The resultant solution was filtered using centrifugation, and the material obtained was dried at a temperature of 65 °C overnight. The obtained powder was annealed at 400 °C. The resulting powder was then annealed at 400 °C in a muffle furnace. Pure MnO_2_ NPs were synthesized in the same procedure, except that we only used manganese(II)nitrate-tetrahydrate (Mn(NO_3_)_2_·4H_2_O). The fabricated NPs were analyzed using various characterization techniques, and all instrument details can be found in our previously reported publication [63].

### 3.2. Nanoparticle Preparation for Biological Studies

For biological experiments, the NP stock was prepared by suspending NPs in ultrapure water (UPW; vehicle) at 1 mg/mL. The suspended NPs were briefly sonicated using a probe sonicator (7 cycles of 10 sec on/10 sec off). DLS analysis is shown in Table 1.

### 3.3. Cell Culture and Viability Studies

The Raw 264.7 cell line (TIB-71) was purchased from the American Type Culture Collection (ATCC, Manassas, VA, USA). Cells were cultivated at 37 °C and 5% CO_2_ using standard aseptic techniques. Cells were grown in Dulbecco’s Modified Eagle’s Medium (DMEM) and supplemented with 10% fetal bovine serum (FBS), 100 U penicillin/mL, and 100 µg streptomycin/mL (Gibco, Waltham, MA, USA).

The viability of cells was estimated via the MTT assay. Cells were grown into 96-well cell culture plates and allowed to develop to 70% confluency. Cells were treated with NPs at a range of concentrations (6.25, 12.5, 25, 50, or 100 µg/mL) for 24 h. The MTT reagent was added to each well, and the plate was shaken for 5 min before incubating at 37 °C for 45 min until color formation. After discarding the supernatants from each well, isopropyl alcohol was added to dissolve the formazan crystals, and the plate was shaken for 10 min. Absorbance in each well was then measured in a Synergy HT system at 570 nm (BioTek, Winooski, VT, USA).

### 3.4. Cell Phagocytosis

Cell phagocytic activity was determined as previously prescribed, based on measuring the engulfed latex beads with a fluorophore-labeled rabbit IgG, as directed by the manufacturer (Cayman, Ann Arbor, MI, USA) [43,64]. In summary, cells were plated in a 24-well plate and cultivated until 80% confluency; the media were then discarded, and the cells were exposed to 6.25 and 12.5 µg/mL of NPs for 2 h. Following this, the cells were washed and treated with new latex bead-containing culture media that delivered particles at a 200:1 cell ratio. The cells were incubated at 37 °C for 4 h before being gently rinsed with a cell-based assay buffer, collected via centrifugation, and resuspended in a cell-based assay buffer. Cells were gated to remove debris or dead cells, and total fluorescence was recorded at 10,000 events per sample using the BD Accuri^TM^ C6 flow cytometer.

### 3.5. Gene Expression

Real-time PCR was used to quantify gene expression via the amplification of mRNA transcripts 7500 Fast RT-PCR (Applied Biosystems, Foster City, CA, USA). The cells were grown into 12-well culture plates to 70% confluency before being exposed to the NPs for 6 h at 12.5 µg/mL. Total RNA was extracted using TRIzol™ LS Reagent (ThermoFisher Scientific, Pleasanton, CA, USA) based on the manufacturer’s protocol. Jenway Genova Nano (Cole-Parmer, Vernon Hills, IL, USA) was used to measure RNA concentration and quality. Using RT Master Mix HY-K0510 (MedChemExpress, South Brunswick Township, NJ, USA) and a Veriti thermocycler (Applied Biosystems, Woburn, MA, USA), mRNA was reverse transcribed into cDNA. Real-time PCR was carried out using an SYBR Green qPCR Master Mix (Low ROX) HY-K0522 (MedChemExpress, NJ, USA), reverse and forward mouse primers (Table 2) for tumor necrosis factor-alpha (*TNF-α*), Interleukin-1 beta (*IL-1β*), and *GAPDH*, which were obtained from integrated DNA technology (USA). The ΔΔC_T_ technique was used to calculate gene expression in comparison to a housekeeping gene (*GAPDH*).

### 3.6. ROS Generation

The quantification of reactive oxygen species (ROS) was measured via dichlorofluorescin diacetate (H2DCFDA) (ThermoFisher Scientific in Waltham, MA, USA). Briefly, Cells were grown into 96-well cell culture plates and allowed to develop to 70% confluency. Cells were treated with NPs within a range of concentrations (1.56–50 µg/mL). The cells were washed 2× with PBS, followed by the addition of H2DCFDA (5 µM, in PBS). This mixture was incubated with the cells for 60 min at a temperature of 37 °C, while ensuring protection from direct light exposure. Fluorescence was quantified via a spectrophotometer with an excitation–emission wavelength of 495–527 nm (Winooski, VT, USA).

### 3.7. Western Blot Analysis

Protein expression was measured by Western blotting. Cells were cultured in 6-well plates to a confluency of 70–80%. The cells were then treated with NPs (12.5 µg/mL) for 24 h. The cells were rinsed 3× with ice-cold PBS and lysed using a RIPA lysis solution (MedChemExpress, NJ, USA) supplemented with a protease inhibitor cocktail (Sigma-Aldrich, Burlington, MA, USA). The collected samples were sonicated and centrifuged at 10,000 rpm for 10 min at 4 °C. The total protein was quantified using the BCA assay (ThermoFisher Scientific, MA, USA). A concentration of 15 μg/mL of protein was loaded onto precast SDS-polyacrylamide gels after boiling with Laemmli Buffer (Bio-Rad, Hercules, CA, USA) for 5 min. The proteins were then transferred to PVDF membranes using electrophoresis. The membranes were blocked with nonfat milk for 1 h, followed by incubation with an Nrf2 primary antibody (ProteinTech’s, Rosemont, IL, USA) overnight, washing, and incubation with a secondary antibody for 1 h at room temperature. Membranes were developed with Pierce Chemiluminescent Substrate. Image J software (https://imagej.net/ij/download.html, accessed on 1 January 2024) was used to quantify the intensity of bands relative to the housekeeping protein (Beta-Actin).

### 3.8. Nanoparticle Internalization

The cellular internalization of Mn was quantified via inductively coupled plasma mass spectrometry (ICP-MS). Briefly, cells were grown on 24-well cell culture plates and allowed to develop to 70% confluency. Cells were then treated with NPs (12.5 µg/mL) for 24 h. Later, samples were pelleted via centrifugation (5000 rpm, 5 min at 4 °C) and washed 3× with ice-cold phosphate-buffered saline (PBS, pH 7.4). The pellet was then dissolved in 10 mL 1% HNO_3_, and NP internalization was measured using an Elan 9000 ICP-MS instrument (Perkin Elmer, Waltham, CT, USA). The process of instrument calibration included the use of a solution containing manganese (Mn) and zinc (Zn) at a concentration of 1 part per billion (ppb). The same standard solution was used for the purpose of optimizing nebulizer gas flow, mass calibration, and resolution. A multi-element internal solution with a concentration of 20 parts per billion (ppb) was used.

### 3.9. Statistical Analysis

All experiments were conducted at least in triplicate (*n* ≥ 3). Data are shown as mean ± standard error of mean (SEM). A *p* value of less than 0.05 (*p <* 0.05) indicates statistical significance. Significant difference between two treatment groups was determined via Student’s *t*-test. Significant differences between more than two treatment groups were determined via one-way analysis of variance (ANOVA) with Tukey’s post hoc testing.

## 4. Conclusions

In conclusion, the main finding of this report indicates that doping with ZnO (1%) has the potential to ameliorate the toxicity of MnO_2_ NPs in Raw 264.7 cells. Although doped NPs induced higher ROS generation and antioxidant responses, they reduced cellular toxicity compared to undoped NPs, as manifested by the reduction in cell viability and cell functional capacity. This suggests that undoped MnO_2_ NPs induced toxicity via ROS-independent mechanisms (e.g., ion release, cellular internalization and direct interaction with and the disruption of organelle function, ROS-independent activation of cellular apoptosis and necrosis, etc.). Future work is needed to delineate the exact molecular mechanisms of toxicity between doped and undoped NPs.

## Figures and Tables

**Table 1:** DLS measurements.

Nanoparticle—Media	Hydrodynamic Size (*d*_h_) (nm)	Zeta Potential (ζ) (mV)	PDI
MnO_2_ NPs—UPW	197 ± 35	−16.2 ± 3.7	0.20
MnO_2_ NPs—DMEM	483 ± 15	−4.8 ± 1	0.25
ZnO-MnO_2_ NPs—UPW	245 ± 7	−20 ± 5.2	0.25
ZnO-MnO_2_ NPs—DMEM	546 ± 30	−5.5 ± 1.8	0.29

UPW: ultrapure water. PDI: polydispersity index.

**Table 2:** List of primers.

Gene	Primer Sequence
*TNF-α*	Forward 5′-ACTGAACTTCGGGGTGATTG-3′ Reverse 5′- GCTTGGTGGTTTGCTACGAC-3′
*IL-1β*	Forward 5′-CTATGGCAACTGTCCCTGAA-3′ Reverse 5′-GGCTTGGAAGCAATCCTTAATC-3′
*GAPDH*	Forward 5′-GGAAAGCTGTGGCGTGAT-3′ Reverse 5′-AAGGTGGAAGAATGGGAGTT-3′

## Data Availability

Data are contained within the article.

## Supplementary Materials

The following supporting information can be downloaded at: https://www.mdpi.com/article/10.3390/ph17020168/s1, Figure S1: Zn content following exposure to nanomaterials. Zn content following treatment with MnO_2_ NPs (grey bars) or ZnO-MnO_2_ NPs (red bars) (12.5 μg/mL) for 24 h. Independent experiments were carried out at least 3 times (*n* ≥ 3). A *p* value of less than 0.05 (*p* < 0.05) indicates statistical significance between control and treatment groups (*). Table S1: Elemental composition of the NPs.
