# Supplementary material for: Synthesis, Characterization, and Toxicity Assessment of Zinc Oxide-Doped Manganese Oxide Nanoparticles in a Macrophage Model
Source: Pharmaceuticals (Basel). 2024 Jan 29;17(2):168. doi: 10.3390/ph17020168 (PMC10892842; doi:10.3390/ph17020168)
Supplement: Supplementary file 1 [file pharmaceuticals-17-00168-s001.zip › pharmaceuticals-2830801-supplementary.pdf]

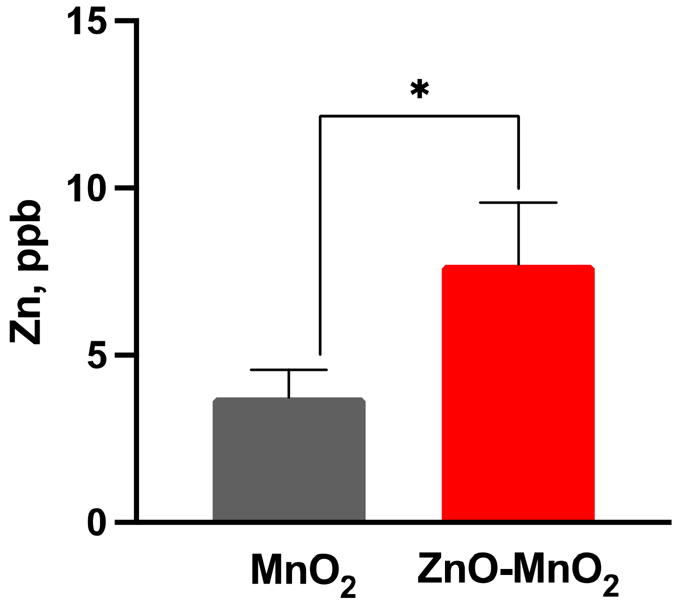

Figure S1. Zn content following exposure to nanomaterials. Zn content following treatment with MnO_2 NPs (grey bars) or ZnO-MnO_2 NPs (red bars) (12.5 µg/ml) for 24h. Independent experiments were carried out at least 3 times (n≥3). A *p* value of less than 0.05 ($p<0.05$) indicates statistical significance between control and treatment groups (*).

Elemental mass (%)	NPs	Elements		
		**Mn**	**O**	**Zn**
	**MnO_2**	68	26.43	
	**ZnO-MnO_2**	61.28	32.47	0.66

Table S1: Elemental composition of the NPs
